# Bacterial response to glucose addition: growth and community structure in seawater microcosms from North Pacific Ocean

**DOI:** 10.1038/s41598-022-27251-2

**Published:** 2023-01-07

**Authors:** Md Nurul Haider, Md Mehedi Iqbal, Masahiko Nishimura, Eiko Ikemoto, Minoru Ijichi, Kazuhiro Kogure

**Affiliations:** 1 Atmosphere and Ocean Research Institute, The University of Tokyo, 5-1-5, Kashiwanoha, Kashiwa-Shi, Chiba, 277-8564 Japan; 2 Faculty of Fisheries, Bangladesh Agricultural University, Mymensingh, 2202 Bangladesh; 3 Department of Biological Sciences, Tokyo Metropolitan University, 1-1 Minami-Osawa, Hachioji-Shi, Tokyo, 192-0397 Japan; 4 University of The Ryukyus, Senbaru, Nishihara, Okinawa, 903-0213 Japan

**Keywords:** Microbiology, Microbial ecology

## Abstract

Onboard microcosm experiments were conducted to assess how bacterial growth pattern and community structure changed by the addition of labile organic compound during the KH-14-2 cruise of R/V *Hakuho Maru* (Atmosphere and Ocean Research Institute, the University of Tokyo and JAMSTEC) in May–June 2014. Seawater samples were collected from the three diversified oceanic environments, Kuroshio Current, North Pacific Sub-polar Gyre (SPG), and North Pacific Sub-tropical Gyre (STG) in the western North Pacific Ocean, filtered, supplemented with glucose, and incubated at 23 ± 1 °C, ~ 4 °C, and 23 ± 1 °C, respectively. Untreated control microcosms were also maintained for all the sample types. Significant increases in cell counts and cell sizes were observed in Kuroshio Current and STG waters, whereas in SPG neither the counts nor the sizes changed, even after 120 h of incubation. At early stages of incubation, the classes Bacteroidia, Alphaproteobacteria, and Gammaproteobacteria were dominant in the Kuroshio Current and SPG samples, while the phyla Cyanobacteria and Proteobacteria in the STG samples. Over incubation periods between 60 and 96 h, some members of the class Gammaproteobacteria gradually dominated within which the genera *Vibrio* and *Alteromonas* became dominant in the Kuroshio Current and STG, respectively. No growth was detected for the microcosms with seawater from SPG, regardless of glucose amendment. It is concluded that depending on the environmental condition, certain different bacterial groups proliferated quickly and modified the community structures. Temperature significantly influenced the growth and succession, and ultimately the community structure of bacteria.

## Introduction

Since marine microorganisms contribute to biodegradation of organic matter leading to the initiation of grazing food web, they are essential for global carbon cycling and nutrient renewal in the ocean^1^. They have functionally specialized and diverse types of metabolism. They spread in all over the oceanic environments, even in those critical to other organisms and are engaged in all the geochemical processes happening in the oceans^2^. Various types of organic matter that are sporadically introduced into the environments are utilized by marine heterotrophic bacteria and considered as a significant factor in controlling bacterial diversity and community structure in marine environment^3^. Then, how do bacteria interact with organic matter is an important question to be answered. Which group of microorganisms utilize what types of organic matter? And how do such processes affect the composition of bacterial community structure and organic matter in marine environments?

The organic matter can be largely categorized into dissolved organic matter (DOM) and particulate organic matter (POM). The former pass through a Whatman GF/F glass fiber filter (pore size approx. 0.7 µm) whereas the latter retained on it^4^. As DOM usually comprise 90% of total organic matter, dissecting its microbial utilization mechanism is critical to understand the carbon cycle in the ocean.

DOM consists of two fractions, i.e., low molecular weight (LMW, < 1 kDa) or monomeric substances, and high molecular weight (HMW, > 1 kDa) or polymeric substances^5^. Since marine bacteria are able to assimilate only LMW DOM (usually < 600 Da)^6–8^, HMW DOM should be first hydrolyzed into smaller units by extracellular enzymes^9,10^. Because the ability of hydrolyzation depends on the possession of the corresponding enzyme^11^, various works have been conducted to investigate which phylogenetic groups are involved in this process. Among the major classes of the phylum Proteobacteria, Alphaproteobacteria was reported to assimilate polymeric substances and monomeric substances^12,13^. The class Betaproteobacteria was found as prominent degraders of the HMW DOM in some cases^5^, while the class of Gammaproteobacteria was also involved in the degradation of HMW DOM^5,12^ and extracellular polymeric substances^13^. The class Gammaproteobacteria tends to be present in the high-nutrient region, while the class Alphaproteobacteria in the low^14^. The phylum Bacteroidota is considered to be specialized in biodegrading the HMW-DOM^15,16^, especially in coastal environment, and they prefer to be attached to marine particles^17^.

On the other hand, relatively few works have been conducted for the utilization of monomeric compounds. Supplementation of glucose and dissolved free amino acid (DFAA) enhanced bacterial production, abundance, and growth rates^18–21^. Culture experiments conducted to assess the importance of resource limitation in controlling bacterial growth, clarified that all samples with glucose showed significant increases in bacterial cell volume^21^. It is expected that any cells possessing specific transport system and metabolic pathway may be able to use such monomeric compounds. However, if there are any particular phylogenetic groups which quickly respond to the addition of monomeric compounds, increase in numbers, and modify the bacterial community structure of various environments. If so, how glucose influence the growth and succession of bacteria in the marine waters? How environmental factor like temperature influence their responds to glucose addition? If such responds and changing pattern is similar among communities in various environments or it varies.

In order to answer these questions, onboard microcosm experiments were conducted using surface seawater collected from three different environments, i.e. Kuroshio Current, North Pacific Sub-polar Gyre (SPG), and North Pacific Sub-tropical Gyre (STG). These three locate off-shore area where influence of terrestrial environments is negligible and indigenous bacterial population and organic compounds are maintained. At SPG and STG, seawater samples at subsurface and chlorophyll maximum layer were also collected, because there may be differences in composition and concentration of DOM and nutrients. It was expected that at SPG, the water column was vertically well mixed, while at STG, vertical mixing is quite limited due to stable stratification. After removal of POM and predators by filtration, glucose was added as a model monomeric compound together with nitrogen (NaNO_3_) and phosphorus (NaH_2_PO_4_) sources. Sugars are vital compounds for most of the heterotrophic bacteria. Among them, glucose as a monomeric substance is generally preferable to others since it can easily be utilized and enhance the production and growth rates of bacteria as much as tenfold^20,21^. Glucose is also known to be most abundantly present monomeric carbohydrate and supporting a considerable part of bacterial production in marine environments^22^. Non-amended control in which bacterial growth is supported by only naturally occurring DOM was also run in parallel. Subsamples were taken for analyzing bacterial biomass and community structures. We hypothesized that depending on the environments, different phylogenetic groups of bacteria respond quickly and increase their relative biomass. To the best of our knowledge, microcosm experiments to assess the insights of bacterial community dynamics had never been conducted using waters from the North Pacific open ocean.

## Materials and methods

### Seawater sample collection

**Figure 1:**
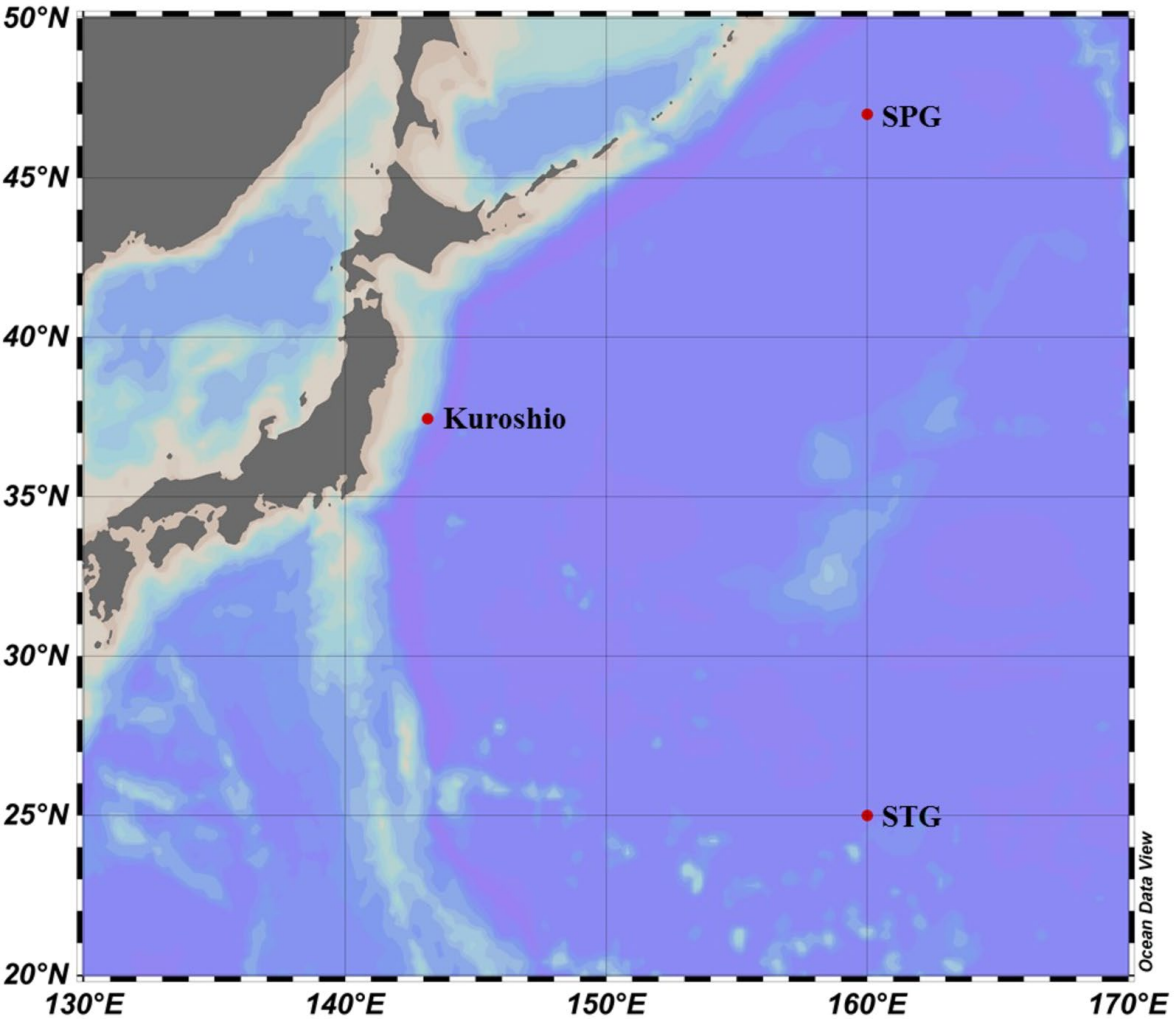
Water samples were collected from three different sampling locations for microcosm experiments: Kuroshio Current, North Pacific Sub-polar Gyre (SPG), and North Pacific Sub-tropical Gyre (STG) in the western North Pacific Ocean. Microcosm experiments were conducted on board during the KH-14-2 cruise in May–June 2014.

Seawater microcosm experiments were conducted onboard during the KH-14-2 cruise of R/V *Hakuho Maru* (Atmosphere and Ocean Research Institute, U of Tokyo and JAMSTEC) in May–June 2014. Surface seawater was collected from Kuroshio Current in the western North Pacific Ocean (37.45°N, 143.15°E, collected on May 23, 2014). Both seawaters of surface and chlorophyll-*a* maximum (Chlo. Max.) were gathered at a location in the North Pacific Sub-polar Gyre (SPG: 47.0°N, 160.0°E) on May 30, 2014, and at a location in the North Pacific Sub-tropical Gyre (STG: 25.0°N, 160.0°E) on June 5, 2014 (Fig. 1).

### Measurement of environmental parameters

The ambient air temperatures were measured at the time of sampling by using a mercurial thermometer. Seawater data (salinity, water temperature, and depth) were obtained using a CTD profiler (SBE 911 plus; Sea-Bird Electronics, Inc., Bellevue, WA, USA). At SPG and STG, concentrations of nitrate-nitrogen (NO_3_-N), nitrite-nitrogen (NO_2_-N), ammonium-nitrogen (NH_4_-N), silicate (SiO_4_) and phosphate-phosphorous (PO_4_-P) were determined using an auto-analyzer (AACS; Bran + Luebbe GmbH, Norderstedt, Germany) according to Armstrong et al*.*^23^.

### Seawater microcosm and subsampling

Microcosm experiments were conducted using rectangular containers made of transparent acrylic plates, the thickness of which was 0.5 cm. The container was 25 cm long, 25 cm wide and 30 cm tall, with an internal capacity of 20 L. The tanks were designed with seaming lids and a subsampling pore of about 3.0 cm diameter. The sampling pore was used during subsampling and kept secured all time to avoid contamination. Seawater samples were taken out from the containers by using a sterilized pipette. The microcosm experiments were made up of five types of seawaters from three oceanic regions (surface water from ~ 5 m depth for all regions, chlorophyll maximum water from 37 and 125 m for SPG and STG, respectively) (Fig. 1).

Seawater samples were pre-filtered twice to remove POM, most of flagellates, and other predatory organisms. Initially through ⌀47 mm Whatman glass microfiber filters, grade GF/A (particle retention, 1.6 µm; GE Healthcare Life Sciences) and then through ⌀47 mm Whatman Nuclepore polycarbonate track-etched membrane (pore size, 0.8 µm; GE Healthcare Life Sciences). Six to eight liters of pre-filtered seawater were transferred into the acrylic containers (length × width × height = 25 cm × 25 cm × 30 cm; capacity 20 l). Before transferring the water, the tanks were carefully and thoroughly wiped with 70% ethanol solution followed by three times washing with ultrapure water. Regardless of the water sources and environmental condition, each microcosm with filtered seawater was amended with a single dose of 20 mgC/l of glucose (C_6_H_12_O_6_, WAKO) together with the supplementation of nitrogen (NaNO_3_, WAKO) and phosphorus (NaH_2_PO_4_⋅2H_2_O, WAKO), at C: N: P ratio = 200:10:1 (supplementary Table 1). The dose (20 mgC/l) of glucose was determined based on the preliminary assessment (data were not published). The calculated amount of all these chemical substances were dissolved in ~ 50 ml seawater (from the tanks), filtered (⌀25 mm syringe filter, 0.20 µm pore size; Advantec Toyo Kaisha Ltd., Tokyo, Japan), added to the microcosm tank, and stirred to mix thoroughly by using a sterilized glass rod. Similarly, the non-amended incubation was initiated simultaneously for the control.

For the microcosms with seawaters from the Kuroshio Current and STG, the containers were kept at room temperature (23 ± 1 °C) in the culture room. For bacterial cell count, cell volume measurement, and bacterial community structure, seawater samples were collected from these microcosms from 0 h up to 60–96 h at 12-h intervals. Microcosms with seawater from SPG were incubated at ~ 4 °C (within a refrigerated chamber), and samples were collected for similar assessment from 0 h up to 96–120 h at 24-h intervals. In all the cases, dark condition (lights switched off) was maintained in the culture room/refrigerated chamber during incubation. For bacterial community structure analyses, ~ 1 L of seawater was filtered directly through a Sterivex-GP pressure filter unit (pore size, 0.22 µm; Merck Millipore, Billerica, MA, USA) using a peristaltic pump. The cell-captured filter units were stored at − 80 °C until further processing.

### Estimation of bacterial cell densities and cell volumes

For the analysis of cell density, collected water samples were fixed in formalin as described previously^24^. Then, 1 ml fixed water sample was filtered onto ⌀25 mm Isopore membrane (pore-size, 0.2 µm; Merck Millipore) and stained with DAPI (4', 6-diamidino-2-phenylindole). Total cell numbers were determined by epifluorescent microscopy^25^. Approximately 40 to 60 images were taken from each filter, and the cells in 10 randomly selected images were visualized and enumerated. Total counts were then done as the average of triplicates for each sample. For cell volume, 1 ml of fixed water samples were filtered onto ⌀25 mm Isopore membrane (pore-size, 0.2 µm; Merck Millipore). Cell images were generated by atomic force microscopy (SPM-9600J2; Shimazu Co. Ltd., Nakagyo-Ku, Kyoto, Japan). From computer-generated images, length (*l*), width (*w*), and height (*l*-*w*) data of about 50 randomly selected cells were acquired for each sub-sample of all sampling periods using the image analysis system supplied by Shimazu Co. Ltd.. The cell volumes were calculated as Vafm^26^.

### DNA extraction and amplicon sequencing of 16S rRNA gene

The double-stranded DNAs were extracted from the preserved Sterivex-GP pressure filter units by Invitrogen Charge Switch Forensic DNA Purification Kits (Thermo Fisher Scientific, Waltham, MA, USA) with a slight modification adding bead-beating using FastGene ZircoPrep Mini (Nippon Genetics Co. Ltd., Bunkyo-Ku, Tokyo, Japan). The bead-beating cell breakage was achieved using a Micro Smash (MS-100R, Tomy Medico., Ltd., Tokyo, Japan) at 5000 rpm and 4 °C for 30 s^27^. The extracted DNAs were then stored at − 20 °C until polymerase chain reaction (PCR) amplification.

The hypervariable V1–V3 region of 16S rRNA gene was amplified by PCR using the forward primer 27F^28^ with multiplex identifiers (MIDs): 5′-CCATCTCATCCCTGCGTGTCTCCGACTCAGXXXXXXXXXXAGAGTTTGATCMTGGCTCAG-3′, and the reverse primer 519R^29^ with adaptor: 5′-CCTATCCCCTGTGTGCCTTGGCAGTCTCAG(GWATTACCGCGGCKGCTG)-3′, where X's represents the sample-specific multiplex identifier-MID^30^. PCR reactions were carried out in 20 µl of the mixture with *TaKaRa Ex Taq* Hot Start Version, which consisted of 2 µl DNA temperate, 13.1 µl distilled water, 0.6 µl of each primer (5 µM), 2 µl 10× *Ex Taq* Buffer (Mg^2+^ plus) (20 mM), 1.6 µl dNTP mixture (2.5 mM each), and 0.1 µl *TaKaRa Ex Taq* HS (5U/µl) (Takara, Kusatsu, Shiga, Japan) in triplicate. Thermal cycling was carried out for a total of 25 cycles as per the following conditions: initial denaturation at 94 °C for 4 min, denaturation at 98 °C for 10 s, annealing at 55 °C for 30 s, elongation at 72 °C for 1 min and final extension at 72 °C for 10 min^24^. After PCR amplification, the presence of the target PCR product was visually confirmed by agarose gel electrophoresis. PCR products were further purified using Agencourt AMPure XP (Beckman Coulter Inc., Brea, CA, USA) according to the 454 Sequencing Amplicon Library Preparation Method Manual (GS Junior Titanium Series 2012). The purified PCR products were then sequenced by a 454 GS Junior platform (Roche Diagnostics, Brandford, CT, USA) according to the manufacturer's instruction for the 454 GS Junior Titanium Series.

### Sequence data accession number

The raw sequence data were deposited in the DDBJ Sequence Read Archive databases under the accession number DRA011683.

### Sequence analyses

Subsequent sequence analyses, quality checking, and arrangement were conducted using the open-sourced MOTHUR program^31^ according to the protocol described in Haider et al*.*^24^. Unique sequences were initially selected, and then similar sequences were clustered and aligned using "Silva.nr_v138_1.align" file as a reference^32^. Sequencing errors were further reduced by screening, filtering, and de-noising through the pre-cluster method^33^. Chimeras were checked and removed using chimera.uchime^34^. Those sequences were subsequently classified using the Ribosomal Database Project (RDP) reference files to remove inactive components such as chloroplasts, mitochondria, etc. Highly qualified sequences were used to generate a distance matrix and clustered assigning operational taxonomic units (OTUs) at 97% identity level^35^. Representative sequences were used for classification by running the MOTHUR program based on the "Silva.nr_v138_1.tax" file. To standardize the number of reads sequenced between samples, they were subsampled to the samples with the fewest reads (5355 reads) using the MOTHUR program based on operational taxonomic unit (OTU) files clustered at 0.03 cut-off levels.

The species richness (were assessed by calculating the Chao1 index) and rarefactions were considered for diversity indices. The Chao1 index^36^ and rarefactions were calculated using the MOTHUR software at OTU definition at a distance of 0.03.

### Statistical analyses

Nonmetric Multidimensional Scaling fitting with environmental parameters (Meta-NMDS) and redundancy analysis (RDA) was performed based on the normalized (rarified) abundance data of the obtained OTUs of each sample to clarify inter-sample diversities of microbial communities associated with different sample groups (including the sample type and sampling location; glucose and other nutrients supplementation etc.). The permutation test was used following the "MASS"^37^ and "Vegan" packages^38^ from R software (R Development Core Team 2012) version 4.1.2. Similarity percentage analysis (SIMPER) was conducted to identify the top 20 most influential OTUs (obtained based on the Bray–Curtis dissimilarity indices) those made the demarcation between the samples using the R software version 4.1.2^38^. After that RDA was also carried out based on their (top 20 most influential OTUs’) relative abundances and environmental features using the “Vegan” package^38^ in R software version 4.1.2 in order to establish relationship between community structure and environmental features. As environmental features, salinity, incubation temperatures, incubation periods (hours of incubation), glucose treatment (treated or non-treated/control), and sampling stations/locations were considered.

To detect taxa with a significant differential abundance among some samples (e.g. SPG surface and chlorophyll maximum), linear discriminant analysis effect size (LEfSe) measurement^39^ was created according to the web-based tool^40^. For LEfSe, the Kruskal–Wallis test by rank was performed to detect the taxa with a significant abundance, followed by LDA to evaluate the effect size of each differentially abundant taxa. Values were considered significant at *p* < 0.05 for both statistical methods. Taxa with markedly increased effect size were defined as those with an LDA score (log10) > 3.

## Results

### Environmental parameters

Sampling locations, air temperature, water temperature, water depth, salinity, nutrient concentrations (NO_3_-N, NO_2_-N, NH_4_-N, SiO_4_, PO_4_-P), and incubation temperatures are shown in Table 1. The air and water temperatures of the studied locations were 11 and 16.6 °C, 3.1 and 3.8 °C, 3.1 and 3.7 °C, 24.5 and 25.9 °C, 24.5 and 18.8 °C, respectively in the Kuroshio Current, SPG surface layer, SPG chlorophyll maximum zone, STG surface layer, and STG chlorophyll maximum zone. At SPG, the values of different parameters were quite similar (*p* = 0.62, two-tail t-Test; at 5% level of significance) between surface (5 m) and chlorophyll maximum (37 m), indicating the vertical mixing in the upper water column. At STG, the values were relatively different (*p* = 0.39, two-tail t-Test; at 5% level of significance) between surface (5 m) and chlorophyll maximum (125 m), suggesting the vertical stratification of the water column. The in-situ (water) temperatures (6.4 °C, 0.2 °C, 0.3 °C and 4.2 °C) were lower than the incubation temperatures compared to those of Kuroshio Current, SPG surface layer, SPG chlorophyll maximum zone, and STG chlorophyll maximum zone, while 2.9 °C higher than the incubation temperature of the STG surface layer. Nutrient assays revealed a big difference in nutrient concentrations between SPG and STG; the waters from the station STG were nutrient-poor. The incubation temperatures of the onboard microcosms were 23 ± 1 °C, ~ 4 °C, and 23 ± 1 °C in the case of Kuroshio Current, SPG, and STG, respectively (Table 1).

**Table 1:** Environmental properties of three water samples used in microcosm experiments. Microcosm experiments were conducted on board during the KH-14-2 cruise in May–June 2014.

Parameters	Kuroshio Current	North Pacific Sub-polar Gyre (SPG)		North Pacific Sub-tropical Gyre (STG)	
	Surface	Surface	Chlo. Max.	Surface	Chlo. Max.
Sampling location	37.45° N 143.15° E	47.0° N 160.0° E		25.0° N 160.0° E	
Air temperature (°C)	11	3.1		24.5	
Water temperature (°C)	16.6	3.8	3.7	25.9	18.8
Sampling depth (m)	5	5	37	5	125
Salinity	34.7	32.9	32.9	35.3	34.9
NO_3_-N (μM)	ND	21.18	21.42	0.05	0.48
NO_2_-N (μM)	ND	0.24	0.23	0.00	0.06
NH_4_-N (μM)	ND	0.13	0.20	0.00	0.04
PO_4_-P (μM)	ND	1.77	1.78	0.00	0.07
SiO_4_ (μM)	ND	35.02	35.31	1.43	2.57
Incubation temp. (°C)	23 ± 1	~ 4		23 ± 1	
In-situ to incubation temp. difference (°C)	+ 6.4	+ 0.2	+ 0.3	− 2.9	+ 4.2

*Chlo. Max.* chlorophyll*-a* maximum, *Salinity* salinity indicated as practical salinity unit, *ND* no data.

### Bacterial cell densities and cell volumes

At initial incubation periods (12 h to 24 h), the cell densities between the glucose-amended and non-treated microcosms were similar (*p* = 0.74, two-tail t-Test; at 5% level of significance). Highly significant differences (*p* < 0.001, two-tail t-Test; at 5% level of significance) was observed later after 36 h of incubation in Kuroshio and STG samples (Table 2). In case of SPG, the density of bacteria increased slowly. In surface seawater, glucose treated microcosms showed higher density than non-treated control at 96 and 120 h. However, in chlorophyll maximum seawater, no difference was noticed between the two. In case of STG, the addition of glucose resulted in marked increase in the density after 36 h in both seawater samples from surface and chlorophyll maximum layer (Table 2).

**Table 2:** Changes of cell density during the shipboard microcosm experiments with the water collected from Kuroshio Current, North Pacific Sub-polar Gyre (SPG), and North Pacific Sub-tropical Gyre (STG) in western North Pacific Ocean during the KH-14-2 cruise in May–June 2014.

Time sampled after the initiation of the culture	Cell density (× 10^5^/ml)									
	Kuroshio Current		North Pacific Sub-polar Gyre (SPG)				North Pacific Sub-tropical Gyre (STG)			
	Surface		Surface		Chlo. Max.		Surface		Chlo. Max.	
	Control	Treated	Control	Treated	Control	Treated	Control	Treated	Control	Treated
0 h (NF)	7.10		6.37		5.95		3.53		4.21	
12 h	6.36	7.72	5.70	5.58	5.88	6.25	3.18	3.61	2.78	2.88
24 h	10.85	13.07	6.14	5.15	6.16	5.24	3.96	4.07	3.20	3.16
36 h	14.81	38.25	ND	ND	ND	ND	2.74	51.74	7.04	45.63
48 h	16.45	84.43	6.89	5.82	6.61	7.49	2.51	68.67	6.06	43.54
60 h	32.22	119.62	ND	ND	ND	ND	3.43	79.62	5.54	50.63
72 h	ND	187.70	7.82	7.14	7.75	7.75	2.57	93.53	4.93	50.21
96 h	ND	ND	10.32	20.13	8.48	8.31	3.10	82.85	5.70	66.92
120 h	ND	ND	4.44	8.36	9.56	10.37	ND	ND	ND	ND

*Chlo. Max.* chlorophyll-*a* maximum, *NF* not filtrated, *ND* no data.

Bacterial growth as cell volume change (mean ± standard deviation) was also measured for each microcosm (Table 3). In case of Kuroshio seawater, the average cell volume in treated microcosm increased by almost 20-fold after 72 h incubation, while much less increase was observed in the control microcosm. In case of SPG, cell volumes were virtually unchanged even after 120 h of incubation in both surface and chlorophyll maximum seawater microcosms. In the STG seawaters, cell volumes were almost similar up to 24 h and increased primarily in the treated microcosms for both surface and chlorophyll maximum water.

**Table 3:** Changes of cell volume (mean ± standard deviation) during the shipboard microcosm experiments with the water collected from the Kuroshio Current, North Pacific Sub-polar Gyre (SPG), and North Pacific Sub-tropical Gyre (STG) in the western North Pacific Ocean during the KH-14-2 cruise in May–June 2014.

Time sampled after the initiation of the culture	Cell volume (mean ± standard deviation) (µm^3^)									
	Kuroshio Current		North Pacific Sub-polar Gyre (SPG)				North Pacific Sub-tropical Gyre (STG)			
	Surface		Surface		Chlo. Max.		Surface		Chlo. Max.	
	Control	Treated	Control	Treated	Control	Treated	Control	Treated	Control	Treated
12 h	0.011 ± 0.009	0.011 ± 0.010	0.010 ± 0.007	0.008 ± 0.009	0.005 ± 0.004	0.006 ± 0.004	0.012 ± 0.018	0.010 ± 0.011	0.007 ± 0.009	0.009 ± 0.008
24 h	0.018 ± 0.029	0.008 ± 0.010	0.010 ± 0.012	0.007 ± 0.007	0.010 ± 0.010	0.008 ± 0.005	0.015 ± 0.016	0.021 ± 4.042	0.019 ± 0.043	0.026 ± 0.043
36 h	0.031 ± 0.044	0.098 ± 0.098	ND	ND	ND	ND	0.034 ± 0.070	0.038 ± 0.066	0.071 ± 0.049	0.071 ± 0.051
48 h	0.011 ± 0.011	0.201 ± 0.211	0.015 ± 0.023	0.014 ± 0.018	0.011 ± 0.015	0.007 ± 0.004	0.012 ± 0.010	0.069 ± 0.062	0.059 ± 0.074	0.098 ± 0.054
60 h	0.080 ± 0.084	0.252 ± 0.275	ND	ND	ND	ND	0.023 ± 0.027	0.091 ± 0.064	0.098 ± 0.071	0.107 ± 0.099
72 h	ND	0.217 ± 0.234	0.009 ± 0.014	0.012 ± 0.033	0.007 ± 0.005	0.009 ± 0.008	0.044 ± 0.094	0.092 ± 0.063	0.071 ± 0.076	0.134 ± 0.121
96 h	ND	ND	0.010 ± 0.008	0.008 ± 0.010	0.007 ± 0.006	0.008 ± 0.007	0.016 ± 0.027	0.127 ± 0.091	0.085 ± 0.077	0.158 ± 0.099
120 h	ND	ND	0.012 ± 0.015	0.008 ± 0.011	0.008 ± 0.007	0.009 ± 0.006	ND	ND	ND	ND

*Chlo. Max.* chlorophyll maximum.

### Succession and modification in bacterial community structure

**Figure 2:**
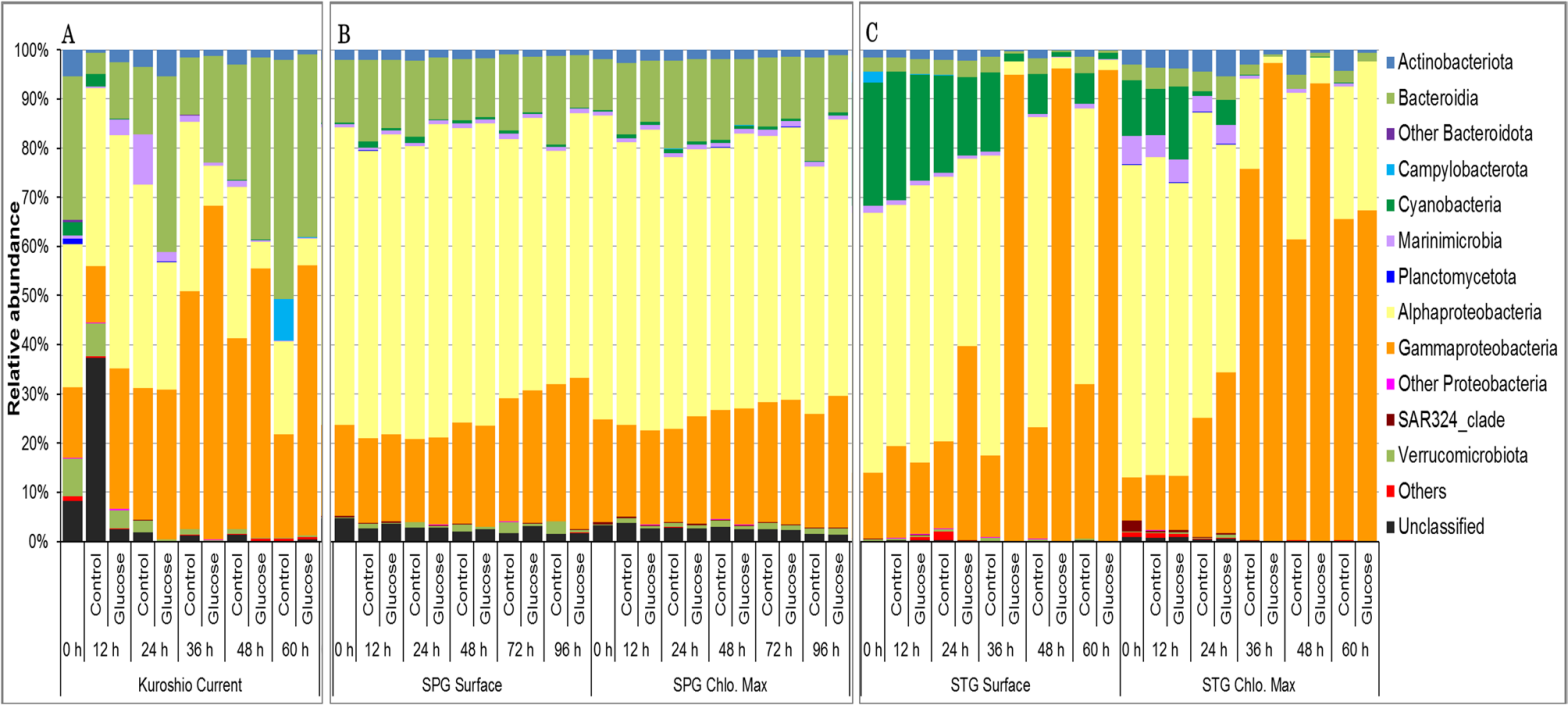
Changes of bacterial community structure during the shipboard microcosm experiments with the water collected from the (**A**) Kuroshio Current, (**B**) North Pacific Sub-polar Gyre (SPG), and (**C**) North Pacific Sub-tropical Gyre (STG) in the western North Pacific Ocean during the KH-14-2 cruise in May–June 2014.

After sequencing all samples, a total of 15,163 operational taxonomic units (OTUs), which were available for molecular phylogenetic analysis, were obtained from 659,424 raw sequence data (supplementary Figs. 1, 2, and 3 showing the rarefaction curves). The community compositions were shown in Fig. 2 at phylum or class levels (e.g., Bacteroidia, Alphaproteobacteria, and so on). The phyla, which contributed less than 1% of the total abundance, were combined and referred to as "Others," and those with no affiliation were referred to as "Unclassified." At the initial stages, the Kuroshio Current and SPG seawaters were dominated by Bacteroidota (class Bacteroidia) and Proteobacteria (classes Alphaproteobacteria and Gammaproteobacteria). In contrast, STG waters were dominated by Cyanobacteria, Marinimicrobia, and Proteobacteria (classes Alphaproteobacteria and Gammaproteobacteria) (Fig. 2). Bacterial communities changed in the treated microcosms collected from Kuroshio Current and STG visibly after 36 h (Fig. 2A,C); the class Gammaproteobacteria proliferated in the treated microcosms. Bacteroidia decreased in early incubation periods of the Kuroshio Current but increased after 48 h incubation in both the treated and control microcosms (Fig. 2A). For the microcosms collected from SPG, minor or no community changes were observed for both the surface and chlorophyll maximum seawaters regardless of the nutritional amendment. In addition, linear discriminant analysis effect size (LEfSe) measurement was conducted to detect any taxa with a significant differential abundance between “control” and “treated” samples of the SPG samples (surface and chlorophyll maximum). No significant (*p* value cutoff *p* < 0.05; LDA score, log10 > 3) features were identified with the given criteria for both SPG-surface and SPG-Chlorophyll maximum (supplementary Table 2).

**Figure 3:**
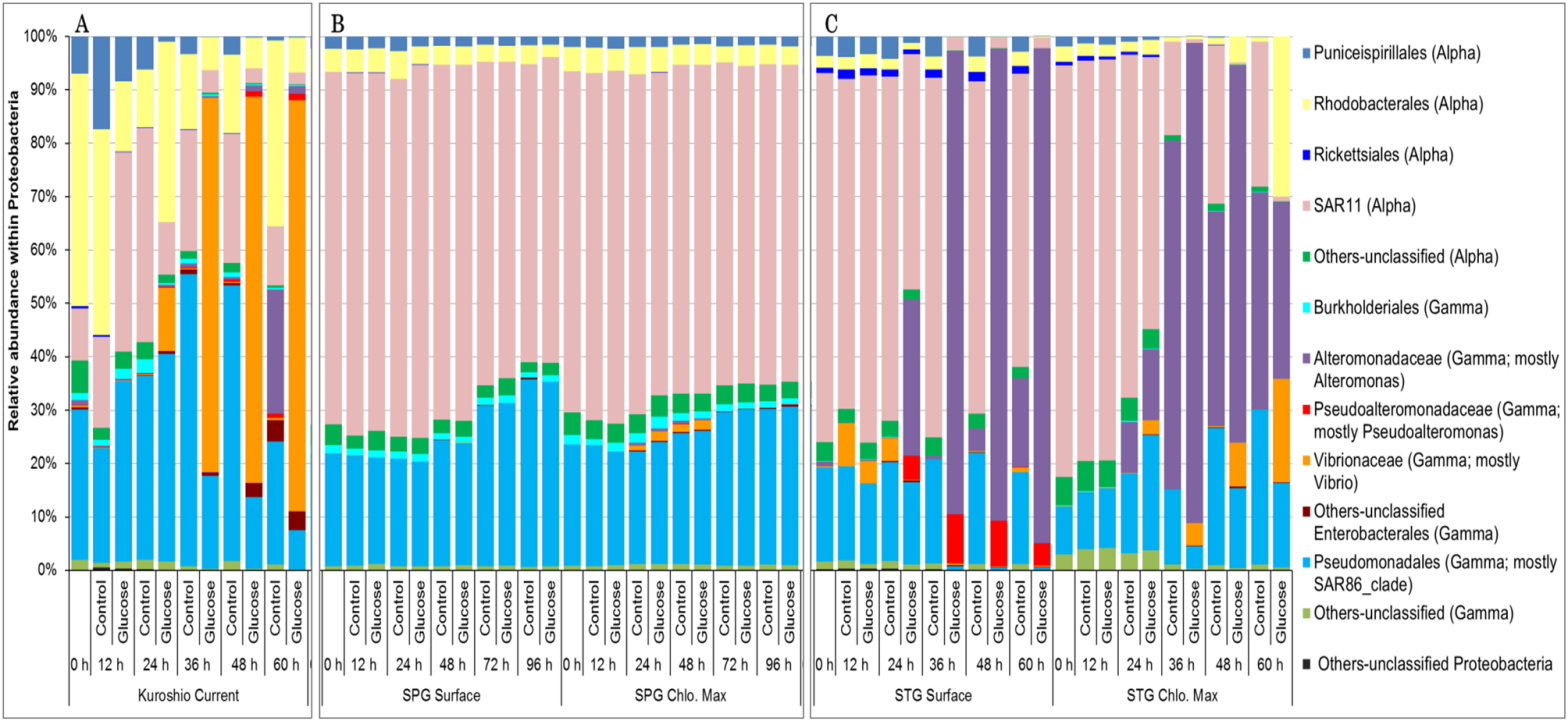
Changes in bacterial community composition within the phylum Proteobacteria at different time intervals in the shipboard microcosm experiments with the water collected from the (**A**) Kuroshio Current, (**B**) North Pacific Sub-polar Gyre (SPG), and (**C**) North Pacific Sub-tropical Gyre (STG) in the western North Pacific Ocean during the KH-14-2 cruise in May–June 2014.

The major groups (classes/orders) in the most dominant phylum, Proteobacteria, were separately shown in Fig. 3. Within the phylum, the composition of the bacterial community gradually changed into a simplified form over time; the class Gammaproteobacteria become dominant (Fig. 3). Within the class Gammaproteobacteria, the genus *Vibrio* (order: Enterobacterales, family: *Vibrionaceae*) were abundant in Kuroshio, while *Alteromonas* (order: Enterobacterales, family: *Alteromonadaceae*) in STG (Fig. 3A,C). Another notable feature of the two major gyre systems (SPG and STG) in the mid-latitudes of the Northern Pacific Ocean is the dominancy of the orders SAR11 (Alphaproteobacteria) and Pseudomonadales (Gammaproteobacteria; mostly SAR86_clade). These two groups were later outnumbered in the case of STG but remained almost unchanged in SPG (Fig. 3B,C). These two groups were also dominant in seawater from Kuroshio; at later periods of incubation, especially the group SAR11 showed a gradual decrease in the treated microcosms (Fig. 3A).

### Species richness and diversity indices

The Chao1 index was calculated for species richness (Table 4) and rarefaction curves were considered for diversity indices (supplementary Figs. 1, 2, and 3). In general, the species richness was relatively higher in unfiltered water samples (samples just after collection), which was reduced after filtration, observed at the initial stages (12 h) of incubation and increased again at 24–48 h. The richness was significantly reduced (*p* = 0.0006 and *t* = 2.26, two-tail t-Test; at 5% level of significance) from the initial period (12 h) to the final periods of incubation (60–96 h) for different microcosms regardless of treatments. However, the Chao1 index values were relatively higher in the control microcosms for almost all the cases at the end of the incubation periods (Table 4). The rarefaction curves also showed that the glucose treated samples were less diverse compared to the controls (supplementary Figs. 1, 2, and 3).

**Table 4:** Changes of species richness (Chao1 index) during the shipboard microcosm experiments with the water collected from the Kuroshio Current, North Pacific Sub-polar Gyre (SPG), and North Pacific Sub-tropical Gyre (STG) in the western North Pacific Ocean during the KH-14-2 cruise in May–June 2014.

Time sampled after the initiation of the culture	Richness (Chao1 Index)									
	Kuroshio Current		North Pacific Sub-polar Gyre (SPG)				North Pacific Sub-tropical Gyre (STG)			
	Surface		Surface		Chlo. Max.		Surface		Chlo. Max.	
	Control	Treated	Control	Treated	Control	Treated	Control	Treated	Control	Treated
0 h (NF)	1052.0		394.7		600.1		632.2		593.1	
12 h	563.4	616.5	438.5	566.2	601.3	622.1	715.0	638.2	594.9	677.3
24 h	500.5	460.2	484.3	541.0	615.3	699.4	589.2	710.0	667.3	648.5
36 h	708.0	349.4	ND	ND	ND	ND	535.3	548.0	553.0	377.8
48 h	528.7	358.5	647.6	542.0	681.3	899.3	562.5	341.7	456.4	340.1
60 h	469.5	426.4	ND	ND	ND	ND	562.5	498.6	387.6	395.1
72 h	ND	ND	560.0	423.0	495.4	397.6	ND	ND	ND	ND
96 h	ND	ND	458.0	420.0	566.8	400.5	ND	ND	ND	ND

*Chlo. Max.* chlorophyll-*a* maximum, *NF* not filtrated, *ND* no data.

### Influence of environmental/incubation parameters on bacterial community structure

**Figure 4:**
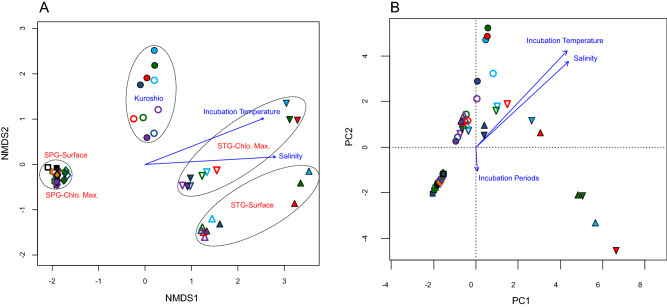
Non-metric Multidimensional Scaling (NMDS) and Redundancy Analysis (RDA) showing the relationship between environmental factors, incubation conditions (periods and temperatures), glucose treatment (treated and control), and bacterial community structure for different sampling stations; Kuroshio Current, North Pacific Sub-polar Gyre (SPG-surface, and SPG-chlorophyll maximum), and North Pacific Sub-tropical Gyre (STG-surface and SPG-chlorophyll maximum). Seawater samples of Kuroshio Current, SPG-surface, SPG-chlorophyll maximum, STG-surface water, and STG-chlorophyll maximum are presented respectively by symbols circle, square, diamond, triangle, and inverted triangle; symbols in purple, dark blue, red, green, light blue, orange, and black indicating incubation periods of 12 h, 24 h, 36 h, 48 h, 60 h, 72 h, and 96 h, respectively; filled symbols are for treated and unfilled symbols are for control samples.

Meta-NMDS and RDA were performed based on the relative abundance data of the obtained OTUs to clarify the influence and/or establish relationship between incubation conditions (time, temperature and glucose treatment) to the bacterial community structure. Meta-NMDS analysis with all the samples showed that they were clustered according to the sampling stations (*r*^2^ = 0.86 and *P* = 0.001, based on 1000 permutations), incubation temperature (*r*^2^ = 0.79 and *P* = 0.001, based on 1000 permutations), and salinity (*r*^2^ = 0.82 and *P* = 0.001, based on 1000 permutations) (Fig. 4A). RDA also showed similar and significant influences of sampling stations (*r*^2^ = 0.54 and *P* = 0.001, based on 1000 permutations), glucose treatment (*r*^2^ = 0.67 and *P* = 0.001, based on 1000 permutations), incubation temperature (*r*^2^ = 0.71 and *P* = 0.001, based on 1000 permutations), and salinity (*r*^2^ = 0.76 and *P* = 0.001, based on 1000 permutations) to the bacterial community structure (Fig. 4B).

**Figure 5:**
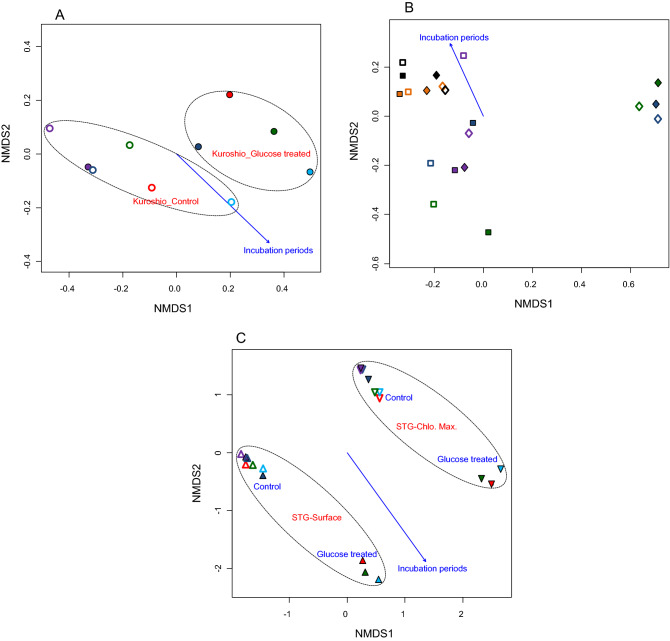
Non-metric Multidimensional Scaling fitting with the incubation conditions (periods and temperatures), and glucose treatment (treated and control) showing the clustering of the samples from each station separately; (**A**) samples of Kuroshio Current; (**B**) samples of North Pacific Sub-polar Gyre, SPG (Surface = surface water, Chlo. Max. = Chlorophyll maximum water); and (**C**) samples of North Pacific Sub-tropical Gyre, STG (Surface = surface water, Chlo. Max. = Chlorophyll maximum water) in the western North Pacific Ocean. Water samples of the Kuroshio Current, SPG-surface, SPG-chlorophyll maximum, STG-surface water, and STG-chlorophyll maximum are presented respectively by the symbols circle, square, diamond, triangle, and inverted triangle; the symbols in purple, dark blue, red, green, light blue, orange, and black indicating incubation periods of 12 h, 24 h, 36 h, 48 h, 60 h, 72 h, and 96 h, respectively; filled symbols are for treated and unfilled symbols are for control samples.

The incubation periods significantly influenced (*r*^2^ = 0.7 and *P* = 0.02, based on 1000 permutations) the clustering of the samples of Kuroshio than that of the glucose treatment (*r*^2^ = 0.3 and *P* = 0.1, based on 1000 permutations) (Fig. 5A). Similar analysis with the samples from SPG also showed significant influence of incubation periods (*r*^2^ = 0.4 and *P* = 0.01, based on 1000 permutations), and seawater source (surface and chlorophyll maximum zone) (*r*^2^ = 0.2 and *P* = 0.02, based on 1000 permutations) with no particular arrangement pattern for the glucose treatment (*r*^2^ = 0.2 and *P* = 0.2, based on 1000 permutations) (Fig. 5B). However, the NMDS with the samples of STG showed clear arrangement according to the water source (*r*^2^ = 0.5 and *P* = 0.001, based on 1000 permutations) and glucose treatment (*r*^2^ = 0.7 and *P* = 0.001, based on 1000 permutations). The incubation periods also significantly influenced (*r*^2^ = 0.3 and *P* = 0.05, based on 1000 permutations) the clustering of the samples of the STG (Fig. 5C).

**Figure 6:**
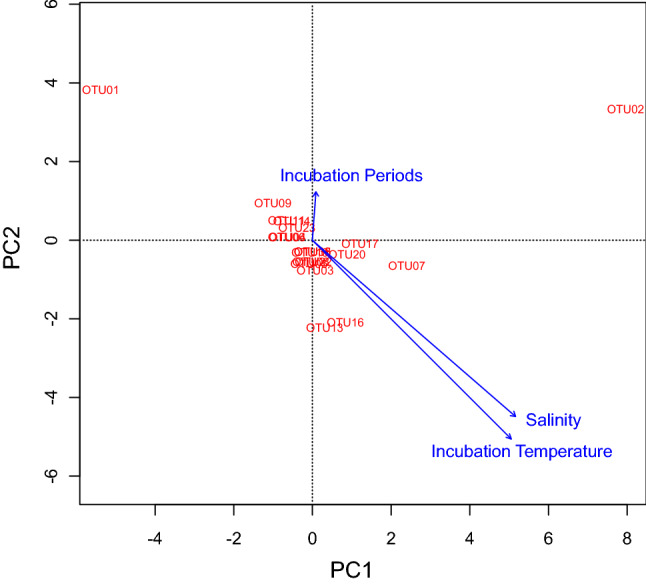
Redundancy analysis (RDA) based on the relative abundances of the most influential 20 OTUs (obtained through similarity percentage, SIMPER analysis) and environmental features. The arrows indicating the influences of the environmental parameters/conditions and the length of the arrows signified the magnitude of the influences. The most influential OTUs were mostly from the phylum Proteobacteria such as—OTU01 = SAR11_clade, OTU02 = *Alteromonas*, OTU07 = *Alteromonas*, OTU16 = *Photobacterium*, OTU13 = *Aurantivirga*, OTU05 = *Prochlorococcus*, OTU08 = SAR11_clade, OTU09 = SAR86_clade, OTU06 = SAR86_clade, OTU20 = *Alcanivorax*, OTU17 = *Alteromonas*, OTU11 = SUP05_cluster, OTU04 = SAR11_clade, OTU14 = NS5_marine_group, OTU19 = SAR11_clade, OTU18 = SAR11_clade, OTU22 = *Rhodobacteraceae*, OTU03 = Vibrio, OTU23 = SAR11_clade, and OTU35 = Bacteria_unclassified. In the sample names K = Kuroshio, PS = SPG surface, PX = SPG Chlorophyll maximum, TS = STG surface, TX = STG Chlorophyll maximum, numeric values = incubation periods, G = glucose treated, and C = control.

Top twenty most influential OTUs, responsible for differentiating the community structure between the samples, were obtained (based on the Bray–Curtis dissimilarity indices) through similarity percentage (SIMPER) analysis (supplementary Table 3). Their (top 20 most influential OTUs) relative abundance data were applied for redundancy analysis (RDA) to establish relationship between community composition and environmental features. The influential OTUs were mostly from the phylum Proteobacteria such as—OTU01 (SAR11_clade (Alphaproteobacteria)), OTU02 (*Alteromonas* (Gammaproteobacteria)), OTU07 (*Alteromonas* (Gammaproteobacteria)), OTU16 (*Photobacterium* (Gammaproteobacteria)), OTU13(*Aurantivirga* (Bacteroidota)), OTU05 (*Prochlorococcus* (Cyanobacteria)), OTU08 (SAR11_clade (Alphaproteobacteria)), OTU09 (SAR86_clade (Gammaproteobacteria)), OTU06 (SAR86_clade (Gammaproteobacteria)), OTU20 (*Alcanivorax* (Gammaproteobacteria)), OTU17 (*Alteromonas* (Gammaproteobacteria)), OTU11 (SUP05_cluster (Gammaproteobacteria)), OTU04 (SAR11_clade (Alphaproteobacteria)), OTU14 (NS5_marine_group (Bacteroidota)), OTU19 (SAR11_clade (Alphaproteobacteria)), OTU18 (SAR11_clade (Alphaproteobacteria)), OTU22 (*Rhodobacteraceae* (Alphaproteobacteria)), OTU03 (*Vibrio* (Gammaproteobacteria)), OTU23 (SAR11_clade (Alphaproteobacteria)), and OTU35 (unclassified bacteria). Based on the relative abundances of these dominant OTUs, their association to environmental factors was examined by redundancy analysis (RDA) (Fig. 6). RDA showed that the samples are separated by treatments (*r*^2^ = 0.66 and *P* = 0.001, based on 1000 permutations) and stations (*r*^2^ = 0.53 and *P* = 0.001, based on 1000 permutations). The incubation temperature (*r*^2^ = 0.76 and *P* = 0.001, based on 1000 permutations) and salinity (*r*^2^ = 0.70 and *P* = 0.001, based on 1000 permutations) also had strong influences on the clustering of the samples, while the incubation periods showed none (*r*^2^ = 0.02 and *P* = 0.62, based on 1000 permutations) (Fig. 6).

## Discussion

In order to clarify how microbial population responds to the addition of monomeric organic compound, seawater microcosm experiments were conducted on board at three different open ocean stations in North Pacific (Fig. 1). Prior to the experiments, sample seawater was filtered through 1.6 µm and 0.8 µm for removing most of POM and flagellates. Therefore, the effects of predation on the community change might be minimum for at least 3–4 days, although the possibility of viral infection is not excluded. The microbial community change in the microcosm without monomeric compound may mimic the actual growth pattern in the environment. The results showed that glucose induced marked increase in biomass for seawater from Kuroshio and STG, whereas no effect was observed for the sample from SPG (Tables 2 and 3). Depending on the station, different bacterial groups responded to the addition (Figs. 2 and 3). Meta-NMDS and RDA showed influences of sampling stations, glucose treatment, incubation temperature and salinity on the bacterial community structure. Bacterial growth was followed in terms of density (Table 2) and cell volume (Table 3). The latter was calculated as Vafm from the images obtained by atomic force microscopy (AFM)^26^. Although AFM has an intrinsic problem to estimate the size accurately, the changing patterns in cell volume during the course of incubation give reliable and meaningful information (Table 3). First, except for SPG, the cell volume tended to increase with time, accompanied with cell number increase. We could not see the case of cellular numerical increase while cell sizes remained small. This may suggest that original small cell in natural environment may not house macromolecular components that ensure active growth. It is not clear how those small cells are actually multiplying. Further research is required to clarify the relationship between the cell volume and physiological state of bacteria in natural environments. Second, neither cell density nor cell volume increased in both treated and non-treated seawater microcosm from SPG. Cherrier et al*.*^41^ reported that in unamended seawater culture experiment in Eastern North Pacific (seawater temperature: 11–13 °C), there was no apparent bacterial increase for 3 days incubation. Kirchman et al*.*^42^ investigated the primary production and bacterial production in the western Arctic Ocean. Bacterial growth rates ranged from 0.023 to 0.144 d^−1^, mostly less than 0.1, indicating that population doubling time may be generally more than a week, which agree with existing literature^41,42^.

The species richness (in Chao 1 index values) and the community structures of the bacteria showed some variations depending on the microcosm conditions. Although the species richness (Chao 1 index values) was fluctuated at the earlier (12 h) to mid-stages (24–48 h), it was significantly reduced (*p* = 0.0006 and *t* = 2.26, two-tail t-Test; at 5% level of significance) at the final stages of incubation (60–96 h), and the Chao 1 index values were relatively higher in the untreated samples (Table 4). The initial (12 h) decrease in species richness was most likely due to the effect of filtration that filtered out the POC together with particle-associated bacteria and predatory organisms. The mid-stage (24–48 h) increase in the species richness was possibly due to the reduction in predation/top-down pressure^43,44^. At the later stages of incubation, especially in the treated samples, some members of the class Gammaproteobacteria, (e.g. genera *Vibrio* and *Alteromonas*) became dominant (Figs. 2 and 3), and some others (e.g. Bacteroidia, Cyanobacteria, Marinimicrobia, SAR11 clade and SAR86 clade, etc.) diminished (Figs. 2 and 3) might be resulting the reduced species richness of bacteria. The community structure of bacteria was reshaped with the periods of incubation especially in the treated microcosms conducted with the seawater from Kuroshio Current and STG (Figs. 2, 3, and 5) (see the details below). Thus, the amendment of organic matter enhances the growth of certain groups and made them dominant, which ultimately reshaped the community structure of the bacteria (Figs. 2 and 3).

The microcosm experiment conducted with the seawater from Kuroshio Current and STG showed a gradual succession in some members of the class Gammaproteobacteria. For example, the genus *Vibrio* became dominant in the Kuroshio Current microcosms while the genus *Alteromonas* in the STG microcosms (Figs. 2 and 3). The members of the class Gammaproteobacteria were usually found relatively abundant at locations with higher nutrients^14^. They seem to be involved in the degradation of HMW DOM^5,12^ and extracellular polymeric substances^13^. Moreover, in the enrichment culture experiments with glucose, the members of Gammaproteobacteria such as *Vibrio* and *Alteromonas* became abundant compared to those of Alphaproteobacteria and Bacteroidia^45^. These findings indicate Gammaproteobacterial preferences to waters rich in organic matter. Under suitable range of temperature, they are able to utilize glucose as a source of carbon and energy; multiply quickly which modify the community structure as observed in the microcosms with water from Kuroshio Current and STG (Figs. 2 and 3). To this end, question may arise why Gammaproteobacteria are less abundant in seawaters even with higher nutrients and why they were found less abundant in most of the previous studies conducted using organic matter. The probable explanation can be, firstly, among different members of the class Gammaproteobacteria, only a few are common in the marine environments^16^, indicating that the members of the Gammaproteobacteria are more sensitive to surrounding conditions compared to those of Bacteroidia and Alphaproteobacteria. They quickly respond to environmental changes and increase or decrease rapidly in the environments. Secondly, the effects of the top-down factors such as predation and viral lyses might be important^46–49^. It is agreed and confirmed by the result of our experiment that Gammaproteobacteira proportionally dominates the microbial community of confined seawater under reduced predation pressure^50^.

Thus, amendment of organic substances modified the community structure of bacteria and the members of the class Gammaproteobacteria usually responded quickly. However, the scenarios were different in the case of the SPG microcosms, in which no apparent growth was observed during the incubation period. The lack of growth (Tables 2 and 3) had ultimately kept a similar community structure of bacteria in both treated and control microcosms of SPG and the SAR11 group constituted more than half of the whole microbial community throughout the period (Figs. 2B and 3B). Felip et al*.*^51^ conducted experiments with nutrient enrichment also reported that incubation at 4 °C resulted in no growth over 48 h, and little or no change later, even when enriched with C, N, and P. The class Alphaproteobacteria prefers monomers; Malmstrom et al*.*^52^ explained this property considering the ubiquity nature of distributions of the clade SAR11 as example. The members of the clade SAR11 are very abundant^53^, involved in the assimilation of LMW DOM. Their limited growth in the microcosm of SPG may be because, firstly, in aquatic environments, bacterial growth and abundance are generally limited by the availability of the dissolved organic matter (DOM) as well as by some other physicochemical properties, especially temperature^54,55^. As the incubation temperature was low (4 °C), the enzymatic activities of the bacteria, the affinity for substrates, and the efficiency of transport proteins of the cell membrane were limited^56^. These might have restricted the utilization and assimilation of the nutrients in SPG microcosms. Secondly, the community structure of bacteria might be already stabilized, and the major groups were adjusted to that nutrient-rich environment (Table 1), which is why the supplementation of nutrients in the microcosms did not show any visible differences^51^. Also, another possibility is the duration of the observation periods (120 h for cell density and 96 h for community structure; Tables 2, 3, Figs. 2B and 3B) of our experiments. Considering that bacterial doubling time in cold environment may exceed 1 week^42^, longer incubation time may be necessary to see the growth. The temperature limitation may be vital for bacterial growth and the nutrient resources only regulate bacterial growth when the temperature remained at adequate level^51^. From these findings, it can be inferred that in the case of SPG, the low temperature restricted the activities of the bacteria, reduced their growth rate, and thus, no recognizable differences were observed in community structure within the incubation periods.

The overall results showed that the members of the class Gammaproteobacteria could react quickly and use low molecular weight substances like glucose at higher incubation temperature (23 ± 1 °C). The dominant genera of this class (Gammaproteobacteria) may vary with environmental conditions. Organic matter triggers the growth of certain groups of bacteria, which are not usually dominant in the natural environments. This growth and succession ultimately change the community structure of bacteria; some environmental factors, such as temperature, significantly affect the growth patterns and ultimately modify the community structure of bacteria.

## Supplementary Information

Supplementary Information.

## Data Availability

The datasets generated during and/or analyzed during the current study are available from the corresponding author on reasonable request.
