# Supplementary material for: Bacterial response to glucose addition: growth and community structure in seawater microcosms from North Pacific Ocean
Source: Sci Rep. 2023 Jan 7;13:341. doi: 10.1038/s41598-022-27251-2 (PMC9825388; doi:10.1038/s41598-022-27251-2)
Supplement: Supplementary file 1 — Supplementary Information. [file 41598_2022_27251_MOESM1_ESM.pdf]

Supplementary data

Supplementary Table 1. Amounts of glucose ($C_6H_{12}O_6$), sodium nitrate ($NaNO_3$), and sodium dihydrogen phosphate ($NaH_2PO_4/2H_2O$) added per liter (l) of water in the treated microcosms. Carbon (C), Nitrogen (N) and phosphorus (P) were added at ratio C: N: P = 200:10:1

**Glucose**		**Sodium Nitrate ($NaNO_3$)**		**Sodium di-hydrogen phosphate ($NaH_2PO_4.2H_2O$)**	
Doses (mg Cl^{-1})	Treated amount (mg l^{-1})	Doses (mg Nl^{-1})	Treated amount (mg l^{-1})	Doses (mg Pl^{-1})	Treated amount (mg l^{-1})
20	50	1	6.07	0.1	0.387
Control			No treatment		

Supplementary Table 2: Linear discriminant analysis (LDA) effect size (LEfSe) among the control and treated samples of the North Pacific Sub-polar Gyre, SPG (surface and chlorophyll maximum). The differences were significant (p <0.05) among classes (Kruskal-Wallis test). The threshold of the logarithmic LDA score was >3.0. A. *Order* level for SPG surface samples, B. *Class* level for SPG surface samples, C. *Order* level for SPG chlorophyll maximum samples, and D. *Class* level for SPG chlorophyll maximum samples.

A

**Orders**	**P values**	**FDR**	**Control samples**	**Treated samples**	**LDA score**
Opitutales (100)	0.009	0.054	218690	43066	-4.94
Flavobacteriales (100)	0.028	0.085	1311700	1028000	-5.15
SAR11_clade (100)	0.251	0.376	5884100	6181800	5.17
Bacteria_unclassified (100)	0.251	0.376	169360	239670	4.55
Pseudomonadales (100)	0.754	0.905	2142000	2238600	4.68
Rhodobacterales (100)	0.917	0.917	274100	268900	-3.42

B

**Classes**	**P values**	**FDR**	**Control samples**	**Treated samples**	**LDA score**
Verrucomicrobiae (100)	0.009	0.045	218690	43066	-4.94
Bacteroidia (100)	0.028	0.071	1311700	1028000	-5.15
Alphaproteobacteria (100)	0.175	0.291	6158200	6450700	5.17
Bacteria_unclassified (100)	0.251	0.313	169360	239670	4.55
Gammaproteobacteria (100)	0.754	0.754	2142000	2238600	4.68

C

**Orders**	**P values**	**FDR**	**Control samples**	**Treated samples**	**LDA score**
Flavobacteriales (100)	0.028	0.141	1119200	750810	-5.27
Pseudomonadales (100)	0.347	0.581	2047200	2252600	5.01
Actinomarinales (100)	0.465	0.581	249540	213410	-4.26
Rhodobacterales (100)	0.465	0.581	236930	264740	4.14
SAR11_clade (100)	0.754	0.754	6347100	6518400	4.93
Flavobacteriales (100)	0.028	0.141	1119200	750810	-5.27

D

**Classes**	**P values**	**FDR**	**Control samples**	**Treated samples**	**LDA score**
Bacteroidia (100)	0.028	0.113	1119200	750810	-5.27
Gammaproteobacteria (100)	0.347	0.620	2047200	2252600	5.01
Acidimicrobiia (100)	0.465	0.620	249540	213410	-4.26
Alphaproteobacteria (100)	0.754	0.754	6584000	6783200	5.00

Supplementary Table 3. Similarity percentage (SIMPER) analysis showing the percentage of specific and cumulative values of the Bray-Curtis dissimilarity indices by the top 20 most influential OTUs responsible for differentiating the community structure between the samples. SIMPER analysis was performed based on the normalized (rarified) abundance data of the obtained OTUs of each sample using the R software version 4.1.2 (Oksanen, 2015).

**SL. No.**	**OTUs**	**Specific Bray-Curtis dissimilarity indices**	**Cumulative Bray-Curtis dissimilarity indices**	**Genera/ Taxa**
1	Otu00001	0.111	0.111	SAR11_clade (Alphaproteobacteria)
2	Otu00002	0.090	0.201	*Alteromonas* (Gammaproteobacteria)
3	Otu00007	0.047	0.249	*Alteromonas* (Gammaproteobacteria)
4	Otu00016	0.033	0.282	*Photobacterium* (Gammaproteobacteria)
5	Otu00013	0.030	0.312	*Aurantivirga* (Bacteroidota)
6	Otu00005	0.028	0.341	*Prochlorococcus* (Cyanobacteria)
7	Otu00008	0.025	0.365	SAR11_clade (Alphaproteobacteria)
8	Otu00009	0.024	0.389	SAR86_clade (Gammaproteobacteria)
9	Otu00006	0.023	0.412	SAR86_clade (Gammaproteobacteria)
10	Otu00020	0.021	0.433	*Alcanivorax* (Gammaproteobacteria)
11	Otu00017	0.020	0.453	*Alteromonas* (Gammaproteobacteria)
12	Otu00011	0.017	0.469	SUP05_cluster (Gammaproteobacteria)
13	Otu00004	0.016	0.485	SAR11_clade (Alphaproteobacteria)
14	Otu00014	0.013	0.498	NS5_marine_group (Bacteroidota)
15	Otu00019	0.012	0.510	SAR11_clade (Alphaproteobacteria)
16	Otu00018	0.012	0.522	SAR11_clade (Alphaproteobacteria)
17	Otu00022	0.011	0.533	*Rhodobacteraceae* (Alphaproteobacteria)
18	Otu00003	0.010	0.543	*Vibrio* (Gammaproteobacteria)
19	Otu00023	0.009	0.553	SAR11_clade (Alphaproteobacteria)
20	Otu00035	0.008	0.560	Bacteria_unclassified

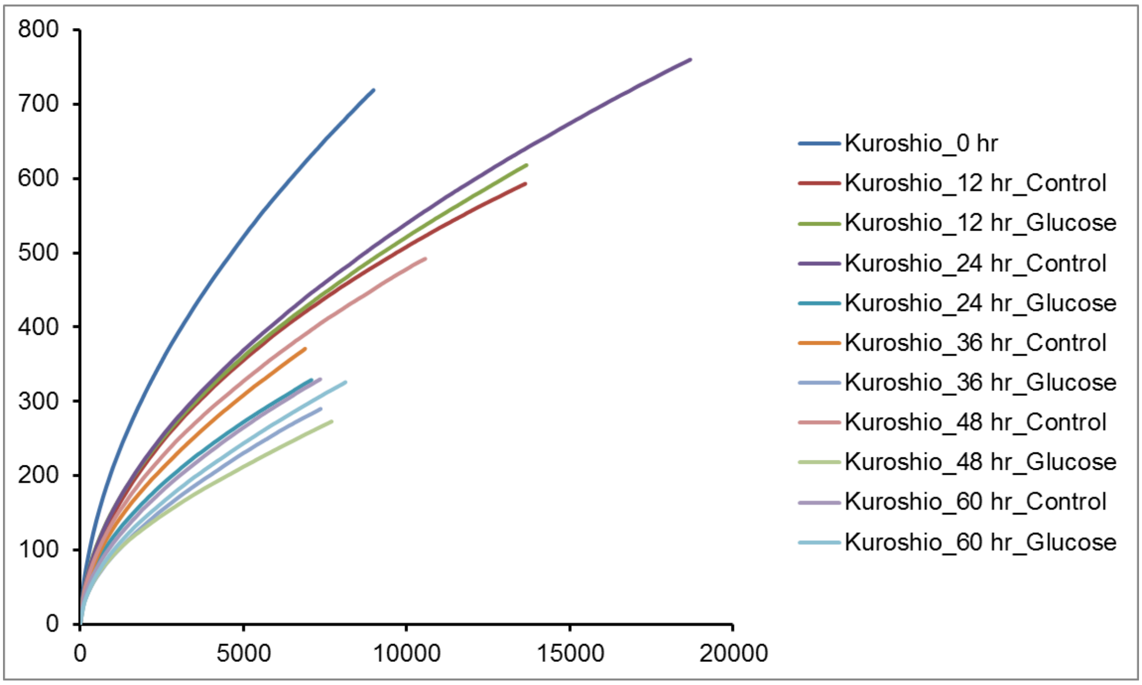

Supplementary Figure 1. Rarefaction curves at different time intervals in the shipboard microcosm experiments with the water collected from the Kuroshio Current during the KH-14-2 cruise in May-June 2014 showing the number of observed OTUs at 0.03 cut-off levels.

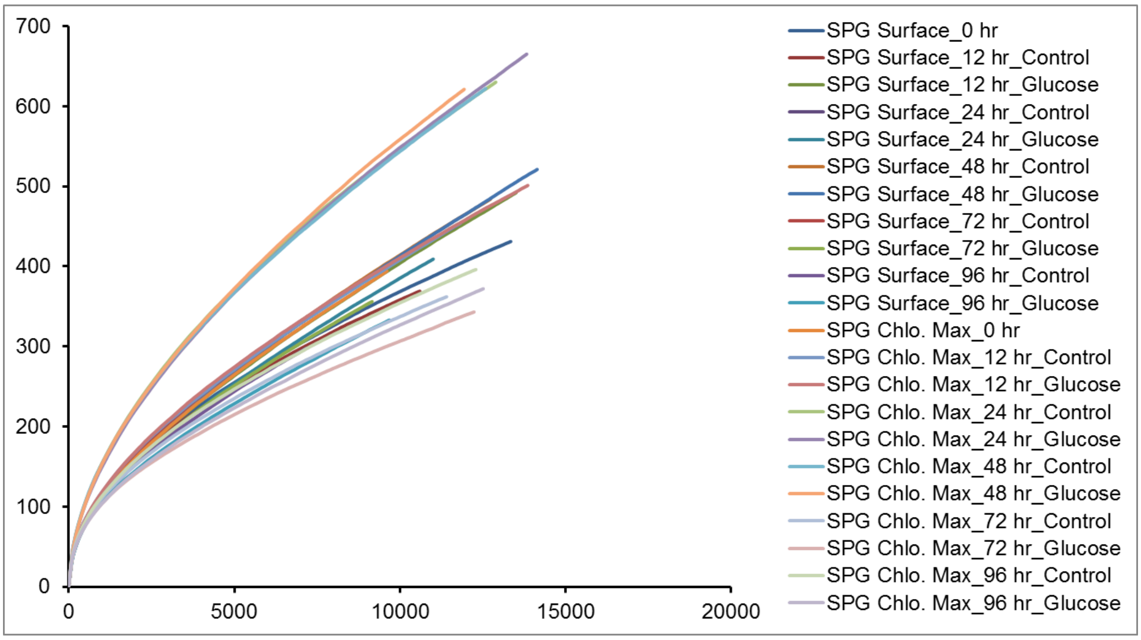

Supplementary Figure 2. Rarefaction curves at different time intervals in the shipboard microcosm experiments with the water collected from the North Pacific Sub-polar Gyre (SPG) in the western North Pacific Ocean during the KH-14-2 cruise in May-June 2014 showing the number of observed OTUs at 0.03 cut-off levels.

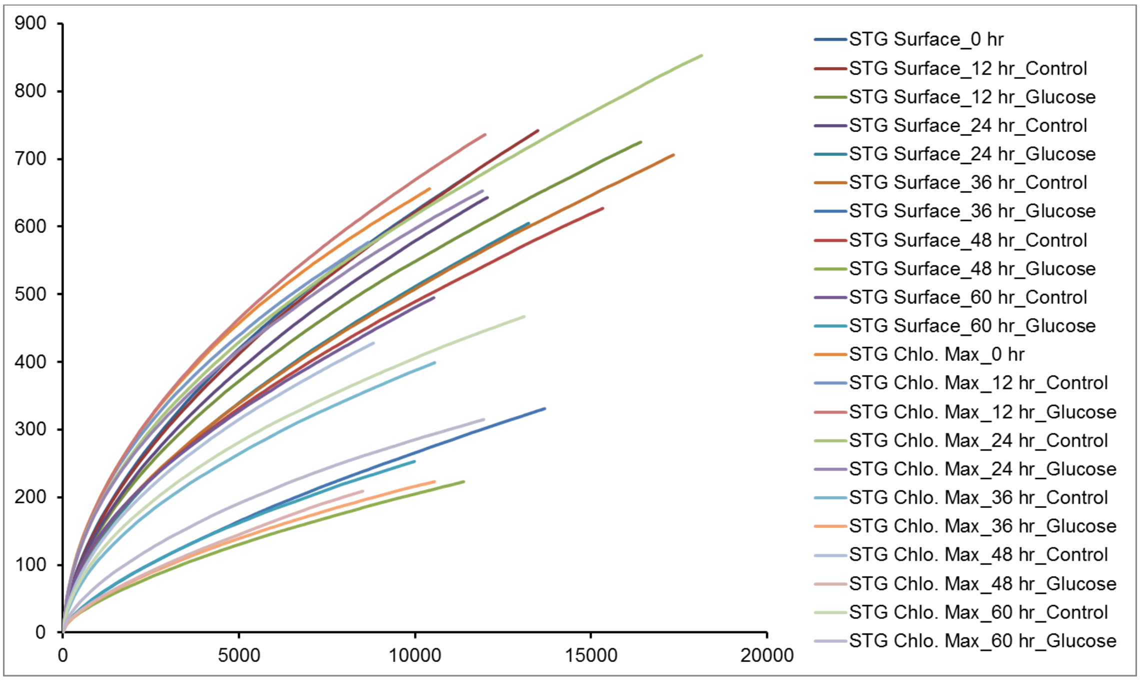

Supplementary Figure 3. Rarefaction curves at different time intervals in the shipboard microcosm experiments with the water collected from the North Pacific Sub-tropical Gyre (STG) in western North Pacific Ocean during the KH-14-2 cruise in May-June 2014 showing the number of observed OTUs at 0.03 cut-off levels.
